# Measuring Restrictiveness in Forensic Mental Health in Germany - Translation and Adaptation of a Questionnaire

**DOI:** 10.1192/j.eurpsy.2022.1955

**Published:** 2022-09-01

**Authors:** P. Walde, B. Völlm

**Affiliations:** Universitätsmedizin Rostock, Klinik Für Forensische Psychiatrie, Rostock, Germany

**Keywords:** Restrictiveness, Psychological Restraint, Questionnaire, Forensic Mental Health

## Abstract

**Introduction:**

A feeling of restrictiveness is often associated with coercive practices, such as seclusion or restraint. In addition to these obvious procedures more subtle practices can also feel restrictive. Its registration and monitoring is of special importance in forensic mental health care since feelings of restrictiveness can lead to adverse events like increased aggression and suicidal intentions.

**Objectives:**

To enable the registration of the experience of restrictiveness in forensic mental health settings in Germany, the Forensic Restrictiveness Questionnaire was translated from English into German.

**Methods:**

Method: We used the TRAPD approach presented by Harkness (2003). This approach combined the expertise of professional translators and clinical experts and enabled adaptation at an early stage. The developed version underwent a cognitive pretest with a small patient sample to check for comprehensibility and interpretation of the questions in line with the original authors intention.

**Results:**

A preliminary translation of the FRQ was developed. Translators combined their expertise from linguistic and clinical practice as well as their knowledge about English and German culture to produce a translation as close as possible to the original questionnaire with necessary adaptations. Remaining uncertainties, e.g., regarding comprehensibility of long phrases or uniform interpretation of certain wordings or questions, were addressed in the cognitive pretest with patients. The version produced can be used for validation.

**Conclusions:**

Conclusion: The TRAPD approach produced a comprehensible and well adapted German translation of the FRQ. This version underwent a cognitive pretest by a small patient sample and is now ready for validation.

**Disclosure:**

No significant relationships.

